# Comparisons of the Prevalence, Severity, and Risk Factors of Dysmenorrhea between Japanese Female Athletes and Non-Athletes in Universities

**DOI:** 10.3390/ijerph19010052

**Published:** 2021-12-21

**Authors:** Reiko Momma, Yoshio Nakata, Akemi Sawai, Maho Takeda, Hiroaki Natsui, Naoki Mukai, Koichi Watanabe

**Affiliations:** 1 Graduate School of Comprehensive Human Sciences, University of Tsukuba, 1-1-1 Tennodai, Tsukuba, Ibaraki 3058574, Japan; monrei015@gmail.com (R.M.); m.clepa13@gmail.com (M.T.); 2 Faculty of Health and Sport Sciences, University of Tsukuba, 1-1-1 Tennodai, Tsukuba, Ibaraki 3058574, Japan; nakata.yoshio.gn@u.tsukuba.ac.jp (Y.N.); mukai.naoki.fu@u.tsukuba.ac.jp (N.M.); 3 Research Institute of Physical Fitness, Japan Women’s College of Physical Education, 8-19-1 Kitakarasuyama, Setagaya, Tokyo 1578565, Japan; sawai.akemi@jwcpe.ac.jp; 4 Faculty of Sports and Health Sciences, Japan Women’s College of Physical Education, 8-19-1 Kitakarasuyama, Setagaya, Tokyo 1578565, Japan; natsui.hiroaki@jwcpe.ac.jp

**Keywords:** menstruation disturbances, menstrual cycle, athletes, women’s health, exercise

## Abstract

This study aimed to investigate the difference in the prevalence, severity, and risk factors of dysmenorrhea between Japanese female athletes and non-athletes in universities. The participants were 18 to 30 years old with no history of a previous pregnancy and/or childbirth. After application of the exclusion criteria, the cohort comprised 605 athletes and 295 non-athletes. An anonymous questionnaire, which included self-reported information on age, height, weight, age at menarche, menstrual cycle days, menstrual duration, dysmenorrhea severity, sleeping hours, dietary habits, exercise habits, training hours, and competition level was administered. Compared with athletes, non-athletes had a higher prevalence of dysmenorrhea (85.6% in athletes, 90.5% in non-athletes, *p* < 0.05); non-athletes also demonstrated increased severity (none/mild 27.8%, moderate 19.3%, and severe 52.9% in athletes; none/mild 21.2%, moderate 17.2%, and severe 61.6% in non-athletes; *p* < 0.05). Factors related to severe dysmenorrhea in athletes included long training hours, early menarche, and prolonged menstrual periods. In non-athletes, short menstrual cycle days and extended menstrual periods were related to severe dysmenorrhea. The prevalence and severity of dysmenorrhea were higher among non-athletes than among athletes; different factors were related to severe dysmenorrhea in these two groups. Thus, different strategies are necessary to manage dysmenorrhea for athletes and non-athletes in universities.

## 1. Introduction

Dysmenorrhea is an important women’s health problem. It is experienced during menstruation and is associated with pain and discomfort such as headaches, abdominal pain, and back pain [1]. There are two types of dysmenorrhea: primary dysmenorrhea, which is caused by excessive prostaglandin secretion without an organic uterine disease, and secondary dysmenorrhea, which is caused by an organic disease of the uterus [2]. Previous studies have demonstrated that the prevalence of dysmenorrhea is approximately 80% among young women; 77.6% among working women (aged 25–55 years) [3]; 83.6% among college students [4]; and 89% among adolescent girls [5]. Moreover, dysmenorrhea is a severe problem in young women because it negatively impacts their lives; for example, it is a cause of absenteeism from school and work and decreased health-related quality of life [6,7].

Bad lifestyle habits may potentially be important risk factors of dysmenorrhea. Short sleeping hours and not having breakfast regularly were associated with moderate-to-severe dysmenorrhea in a previous study [8]. In addition, caffeine consumption [9], alcohol consumption, and smoking [10] were also associated with dysmenorrhea. Moreover, mental stress [11,12,13] and a lack of exercise [14] were related to the severity of dysmenorrhea. Therefore, lifestyle changes may be a potential strategy to manage dysmenorrhea. 

Armor et al. reported that dysmenorrhea lowered athletic performance during training and competitions [15]. Another study showed that the dysmenorrhea pain score was higher in athletes than in sedentary students [16]. Additionally, an interview-based study reported that menstruation-related symptoms reduce athletic performance in athletes [17]. In a previous study, athletes had a lower prevalence of dysmenorrhea than non-athletes (39.44% in athletes and 43.88% in non-athletes), although the difference was not significant [18]. An additional study showed that exercise can reduce dysmenorrhea [14]; however, the participants in this study were women with no exercise habits. Research on dysmenorrhea in athletes and non-athletes has therefore not yielded consistent results.

To address this issue, the present study aimed to investigate the difference in the prevalence, severity, and risk factors of dysmenorrhea between Japanese female athletes and non-athletes in universities. The present study hypothesized that athletes show an increased prevalence of severe dysmenorrhea relative to non-athletes and that different factors are associated with severe dysmenorrhea between these two groups of women.

## 2. Materials and Methods

### 2.1. Study Design

We conducted a cross-sectional, anonymous questionnaire survey administered from October 2019 to March 2020. The participants were recruited using a snowball sampling method, and all individuals consented to participating in this study. The Ethics Review Board of the Faculty of Health and Sport Sciences at the University of Tsukuba approved the study protocol (approval number: 19–85) on 19 September 2019.

### 2.2. Participants

**Figure 1:**
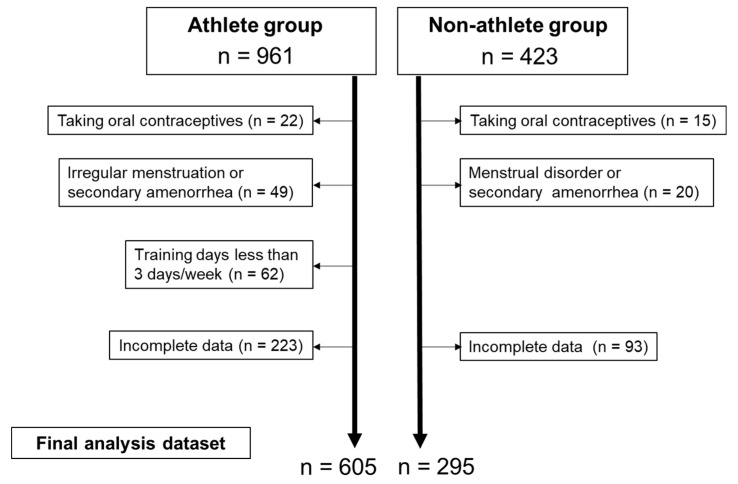
Participant flow diagram.

Our cohort of participants included 961 athletes and 423 non-athletes who were recruited with the help of faculty members from six Japanese universities located in Tokyo (three universities), Ibaraki (two universities), Chiba (one university), and Okayama (one university). The athlete group consisted of university students who majored in physical education or sports science and/or who belonged to athletic clubs. The non-athlete group consisted of university students who majored in subjects other than sports science, such as nutrition and nursing, and/or those who did not participate in athletic competitions, such as managers of athletic clubs. This study included women who were aged 18 to 30 years, those who had never been pregnant and/or given birth, those who did not take oral contraceptives, and those who did not have irregular menstruation or secondary amenorrhea. University athletes were defined as those who belonged to an athletic club (not a recreational club), participated in competitions on a regular basis, and trained at least 3 days per week. As shown in Figure 1, 356 women in the athlete group and 126 women in the non-athlete group were excluded owing to the following exclusion criteria: those taking oral contraceptives (n = 22 and n = 15 in the athlete and non-athlete groups, respectively), those with irregular menstruation or secondary amenorrhea (n = 49 and n = 20, respectively), those who trained less than 3 days a week (n = 62 in the athlete group), and those with incomplete data (n = 223 and n = 93, respectively). The final analysis dataset comprised data from 605 athletes and 295 non-athletes. The athletes played basketball (n = 98), track and field (n = 88), lacrosse (n = 62), handball (n = 62), volleyball (n = 44), soccer (n = 33), rhythmic gymnastics (n = 32), dance (n = 32), softball (n = 27), kendo (n = 23), judo (n = 23), swimming (n = 19), badminton (n = 14), baseball (n = 13), tennis (n = 12), cheerleading (n = 9), gymnastics (n = 6), wheel gymnastics (n = 5), and wrestling (n = 3).

### 2.3. Questionnaire

A questionnaire that included questions related to age, height, weight, age at menarche, menstrual cycle days, menstrual duration, dysmenorrhea severity (none: 0 to heavy pain: 10), sleeping hours, dietary habits (skipping meals), exercise habits (in non-athletes), training hours (per week), and competition level (1: international, 2: national, 3: regional, 4: prefectural, 5: other, in athletes) was prepared. Body mass index (BMI) was calculated using the following formula: weight (kg) divided by the square of height (m^2^). The following question was asked about the prevalence and severity of dysmenorrhea. “What is the degree of pain you experience during menstruation? Please circle the number between 0 and 10 that is reflective of the pain you experience.” Those with a severity score of ≥1 were defined as having dysmenorrhea. With reference to a previous study [19], the severity of dysmenorrhea was classified into three categories, namely, none/mild (0 to 3), moderate (4 to 6), and severe (7 to 10). Additionally, gynecological age was calculated by subtracting the age at menarche from the calendar age [20]. The severity of dysmenorrhea was compared between competition levels or sport types in university athletes.

### 2.4. Statistical Analysis

Data were analyzed using SPSS version 26 (SPSS Inc., Chicago, IL, USA). The Kolmogorov–Smirnov normality test was used to examine normality. Because all variables were not normally distributed, the Mann–Whitney test was conducted to compare the characteristics of the participants, and the chi-square test was conducted to compare the prevalence and severity of dysmenorrhea between athletes and non-athletes, between sport types, and between competition levels. The Kruskal–Wallis test was used to compare the characteristics of the participants according to the severity of dysmenorrhea in athletes and non-athletes, followed by a Bonferroni post hoc test. The effect sizes were calculated and expressed as ES [21]. A logistic regression model was used to identify risk factors for severe dysmenorrhea (severe or not); the severity of dysmenorrhea was the dependent variable in the athlete and non-athlete groups. Independent variables were age, BMI, sleeping hours, skipping meals, age at menarche, menstrual cycle, menstrual period, training hours (in athletes), competition level (in athletes), and exercise hours (in non-athletes). The odds ratio and 95% confidence interval (95% CI) were calculated for each variable. Data are expressed as median (interquartile range) or frequency (%).

## 3. Results

### 3.1. Participant Characteristics

As shown in Table 1, some characteristics differed significantly between athletes and non-athletes. Athletes were taller, heavier, and higher in BMI and had shorter menstrual periods, a younger gynecological age, and longer sleeping hours than non-athletes. The prevalence of dysmenorrhea was significantly higher in non-athletes than in athletes (*p* = 0.04, ES = 0.07). 

### 3.2. Dysmenorrhea Severity

**Figure 2:**
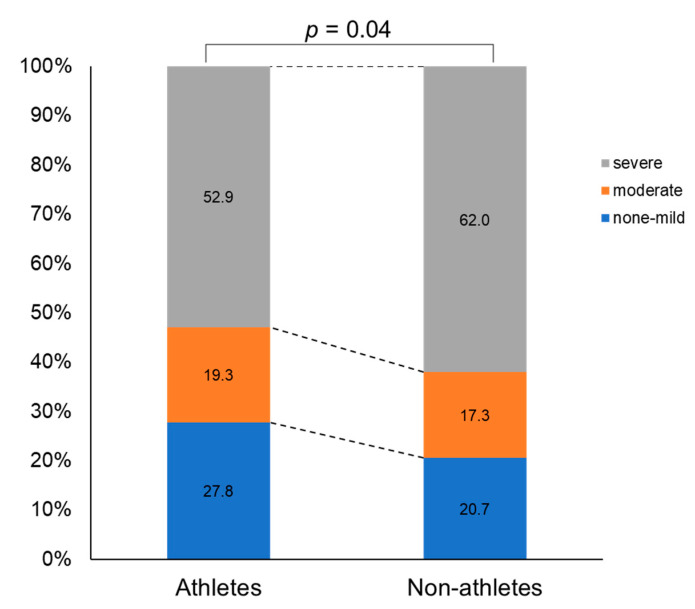
Severity of dysmenorrhea in athletes and non-athletes.

As shown in Figure 2, the severity of dysmenorrhea differed significantly between athletes and non-athletes (*p* = 0.04, ES = 0.09). Dysmenorrhea was shown to be more severe in non-athletes (none/mild 21.2%, moderate 17.2%, and severe 61.6%) than in athletes (none/mild 27.8%, moderate 19.3%, and severe 52.9%).

Table 2 and Table 3 present characteristics by the severity of dysmenorrhea in athletes and non-athletes, respectively. In athletes, there were significant differences in age, age at menarche, menstrual period, and gynecological age between the dysmenorrhea severity groups. However, there were no differences in these variables between competition levels or between sport types. In non-athletes, there were no significant differences in these variables between the dysmenorrhea severity groups. 

### 3.3. Factors Related to Severe Dysmenorrhea

Table 4 and Table 5 illustrate the coefficients at 95% CIs generated from the logistic regression model using dysmenorrhea severity (severe or not) as the dependent variable in athletes and non-athletes, respectively. In athletes, long training hours, early menarche, and a long menstrual period were significantly related to severe dysmenorrhea (Table 4). In non-athletes, a short menstrual cycle and a long menstrual period were significantly related to severe dysmenorrhea (Table 5).

## 4. Discussion

The present study investigated the difference in the prevalence, severity, and risk factors of dysmenorrhea between Japanese female athletes and non-athletes in universities. The prevalence of dysmenorrhea was higher in non-athletes (90.5%) than in athletes (85.6%) (*p* = 0.04, ES = 0.07). Furthermore, the severity of dysmenorrhea was higher in non-athletes than in athletes (*p* = 0.04, ES = 0.09). Although the effect sizes were small, significances were observed. The factors associated with severe dysmenorrhea were different between athletes and non-athletes. As mentioned earlier, long training hours, early menarche, and long menstrual periods were significant risk factors among athletes, while short menstrual cycles and long menstrual periods were shown to be significant risk factors among non-athletes. Therefore, different strategies may be necessary to address severe dysmenorrhea in athletes and non-athletes in universities.

Most previous studies have reported the prevalence of dysmenorrhea among the general population. Polat et al. reported that the prevalence of dysmenorrhea among adult university students in Turkey was 87.8% [22]. In contrast, Ortiz et al. reported a prevalence of 48.4% among Mexican high school students [23]. This inconsistency is partly due to the different definitions of dysmenorrhea. The former study defined dysmenorrhea as having pain during menstruation; the latter defined dysmenorrhea as having painful menstruation for the past 3 months. It is necessary to focus on this point when interpreting the prevalence of dysmenorrhea. The definition from the former study was used in the present study, and the prevalence of dysmenorrhea was similar in both [22].

Few previous studies have reported the prevalence of dysmenorrhea among athletes. Homai et al. compared the prevalence of dysmenorrhea between athletes and non-athletes (39.44% in athletes and 43.88% in non-athletes) [16]. However, they used a different definition of dysmenorrhea. The present study showed that the prevalence of dysmenorrhea was higher in non-athletes (90.5%) than in athletes (85.6%). While the prevalence of dysmenorrhea was much higher in the present study than in the previous study, the rank relationship noted in the present study was comparable to that reported in the previous study [16]. 

Many previous studies have reported the severity of dysmenorrhea among the general population of women; however, few previous studies compared differences in severity between athletes and non-athletes. Some observational studies reported that women without exercise habits had a high prevalence and severity of dysmenorrhea [8,14,24]. Some intervention studies demonstrated that an exercise intervention improved the severity of dysmenorrhea in sedentary women [25,26,27,28]. Therefore, exercise might be a potential strategy to manage dysmenorrhea in the general population of women. 

However, very frequent training may be a risk factor for severe dysmenorrhea in athletes. Czajkowska et al. reported that premenstrual syndrome (PMS) might worsen in athletes due to high-intensity training and an extended competition history [29]. A previous study showed a correlation between the severity of PMS and the severity of dysmenorrhea [30]. Therefore, the present study hypothesized that the prevalence of severe dysmenorrhea is higher in athletes owing to consistent, high-intensity training. However, in the present study, the prevalence of severe dysmenorrhea was shown to be higher in non-athletes (61.6%) than in athletes (52.9%), which is not in line with the initial hypothesis. In addition, there was no difference in the severity of dysmenorrhea between competition levels or sport types in the present study. Further studies are necessary to be conducted in different populations.

The present study also examined the factors related to severe dysmenorrhea in athletes and non-athletes. In athletes, long training duration was a risk factor for severe dysmenorrhea, and this finding is similar to that of a previous study that reported that long training hours are associated with PMS [29]. Although the prevalence of severe dysmenorrhea was lower in athletes than in non-athletes, frequent training may be a risk factor for severe dysmenorrhea. Low-intensity exercises, such as yoga and Pilates, are thought to be beneficial for improving dysmenorrhea because it lowers the levels of cortisol, which in turn inhibits prostaglandin synthesis [31,32]. However, prolonged high-intensity exercise, which is performed by athletes, may increase the levels of inflammatory cytokines, which in turn may increase prostaglandin synthesis and increase the severity of dysmenorrhea [33]. Therefore, the management of training hours might be a crucial factor in controlling dysmenorrhea in athletes.

Long menstrual periods were a common risk factor for dysmenorrhea in university athletes and non-athletes. This result was consistent with those of previous studies conducted on the general population [23,34,35]. In non-athletes, short menstrual cycles were shown to be an important risk factor for dysmenorrhea, while, in contrast to previous studies, exercise habits were not [8,14,24]. Although the risk factors in athletes and non-athletes were examined separately, the previous studies may have included both athletes and non-athletes in the study population. Therefore, the difference in study designs and study populations might have resulted in different findings in these studies. 

There were some limitations in this study. First, the study participants were not a representative sample. Athlete and non-athlete participants were recruited separately. Therefore, the overall sample included in this study could not be analyzed. Second, the participants were recruited from a limited number of universities, and the participants were pursuing studies in physical education, nursing, or nutrition. In addition, we enrolled athletes from many sports in this study. Third, self-reported data were collected using a questionnaire, which contained questions that required participants to recollect events that had happened in the past; this might have led to recall bias. Fourth, primary dysmenorrhea was not differentiated from secondary dysmenorrhea. The causes are different: primary dysmenorrhea is caused by prostaglandins and secondary dysmenorrhea is caused by an organic disease. As the causative mechanisms of primary and secondary dysmenorrhea are different, future studies involving the collection of the history of gynecological consultations and previous medical history are needed. In addition, the diseases that may cause dysmenorrhea were not investigated. Fifth, the validity and reliability of the questionnaires were not tested. Sixth, a detailed survey on the nutritional status of the participants was not conducted. Thus, caution is necessary when generalizing the results of this study.

## 5. Conclusions

The present study compared the prevalence and severity of dysmenorrhea between female university athletes and non-athletes in Japanese universities and investigated the risk factors. The prevalence and severity of dysmenorrhea were higher in non-athletes than in athletes. The risk factors for severe dysmenorrhea were long training hours, early menarche, and long menstrual periods in athletes. In contrast, short menstrual cycles and long menstrual periods were shown to be significant risk factors in non-athletes. Therefore, different strategies may be necessary to address dysmenorrhea in athletes and non-athletes in universities.

## Figures and Tables

**Table 1:** The characteristics of the study participants.

	Athletes (n = 605)		Non-Athletes (n = 295)		*p*	ES
Age (years)	20.0	[19.0–21.0]	20.0	[19.0–21.0]	0.82	0.01
Height (cm)	161.0	[157.0–165.0]	158.0	[155.0–162.0]	<0.01	0.23
Weight (kg)	55.5	[52.0–60.0]	50.0	[47.0–54.0]	<0.01	0.40
BMI (kg/m²)	21.5	[20.3–22.8]	19.8	[18.8–21.3]	<0.01	0.34
Sleeping hours (hours)	7.0	[6.0–7.8]	6.0	[5.5–7.0]	<0.01	0.19
Skipping meals (yes, %)	124	(20.5%)	76	(25.8%)	0.07	0.06
Age at menarche (years)	13.0	[12.0–14.0]	12.0	[11.0–14.0]	<0.01	0.20
Menstrual cycle (days)	30.0	[28.0–30.0]	30.0	[28.0–31.0]	0.03	0.07
Menstrual period (days)	5.0	[5.0–7.0]	6.0	[5.0–7.0]	<0.01	0.10
Gynecological age (years)	7.0	[6.0–8.5]	8.0	[6.0–10.0]	<0.01	0.16
Prevalence of dysmenorrhea (yes, %)	518	(85.6%)	267	(90.5%)	0.04	0.07
Training/exercise hours (hours/week)	18.0	[12.5–24.0]	0.0	[0.0–0.0]	<0.01	0.37
Competition level (n, %)						
International	49	(8.1%)				
National	323	(53.4%)				
Regional	142	(23.5%)				
Prefectural	75	(12.4%)				
Other	16	(2.6%)				

Data are expressed as median [interquartile range] or frequency (%). BMI, body mass index; ES, effect size.

**Table 2:** Characteristics by severity of dysmenorrhea in athletes.

	No/Mild (n = 168)		Medium (n = 117)		Severe (n = 320)		ES	
Age (years)	20.0	[19.0–21.0]	20.0	[19.0–21.0]	20.0	[20.0–21.0]	0.02	^#^
Height (cm)	161.0	[157.5–166.0]	160.2	[155.9–164.2]	161.0	[157.0–165.0]	<0.01	
Weight (kg)	55.1	[51.0–60.0]	55.0	[52.0–60.0]	56.0	[52.0–61.0]	<0.01	
BMI (kg/m^2^)	21.4	[20.1–22.6]	21.3	[20.2–22.8]	21.6	[20.4–22.9]	<0.01	
Sleeping hours (hours)	6.8	[6.0–7.5]	7.0	[6.1–7.9]	7.0	[6.0–7.8]	<0.01	
Skipping meals (yes, %)	30	(17.9%)	18	(15.4%)	76	(23.8%)	0.09	
Age at menarche (years)	14.0	[12.0–15.0]	13.0	[12.0–14.0]	13.0	[12.0–14.0]	0.03	^#^
Menstrual cycle (days)	30.0	[28.0–30.0]	30.0	[28.0–30.0]	30.0	[28.0–30.0]	<0.01	
Menstrual period (days)	5.0	[4.0–6.0]	5.0	[4.0–6.0]	6.0	[5.0–7.0]	0.03	^#$^
Gynecological age (years)	7.0	[5.0–8.0]	7.0	[6.0–9.0]	7.0	[6.0–9.0]	0.05	*^#^
Training hours(hours/week)	18.0	[12.0–24.0]	18.0	[12.3–21.0]	18.0	[12.5–24.0]	<0.01	
Competition level (n, %)							0.10	
International	12	(7.1%)	12	(10.3%)	25	(7.8%)		
National	90	(53.6%)	64	(54.7%)	169	(52.8%)		
Regional	46	(27.4%)	23	(19.7%)	73	(22.8%)		
Prefectural	15	(8.9%)	14	(12.0%)	46	(14.4%)		
Other	5	(3.0%)	4	(3.4%)	7	(2.2%)		

Data are expressed as median [interquartile range] or frequency (%). *: no/mild vs. medium (*p* < 0.01), #: no/mild vs. severe (*p* < 0.01), $: medium vs. severe (*p* < 0.05). BMI, body mass index; ES, effect size.

**Table 3:** Characteristics by severity of dysmenorrhea in non-athletes.

	No/Mild (n = 61)		Medium (n = 51)		Severe (n = 183)		ES
Age (years)	20.0	[19.0–21.0]	20.0	[19.0–22.0]	20.0	[19.0–21.0]	0.01
Height (cm)	158.0	[153.6–161.1]	158.7	[155.4–161.0]	158.0	[155.0–162.0]	<0.01
Weight (kg)	50.0	[48.0–54.0]	51.0	[47.8–54.0]	50.0	[47.0–54.2]	<0.01
BMI (kg/m^2^)	20.4	[19.1–21.4]	20.0	[19.0–21.0]	19.6	[18.7–21.3]	0.01
Sleeping hours (hours)	6.0	[5.1–7.0]	6.5	[5.8–7.5]	6.0	[5.5–7.0]	<0.01
Skipping meals (yes, %)	14	(23.0%)	14	(27.5%)	48	(26.2%)	0.03
Age at menarche (years)	13.0	[11.0–14.0]	12.0	[12.0–14.0]	12.0	[11.0–13.0]	0.01
Menstrual cycle (days)	30.0	[28.0–31.0]	30.0	[28.0–35.0]	30.0	[28.0–30.0]	0.01
Menstrual period (days)	5.0	[4.8–7.0]	5.0	[5.0–6.0]	6.0	[5.0–7.0]	0.03
Gynecological age (years)	8.0	[6.0–9.0]	8.0	[6.0–10.0]	8.0	[7.0–10.0]	0.01
Exercise hours (hours/week)	0.0	[0.0–0.3]	0.0	[0.0–0.0]	0.0	[0.0–0.0]	0.01

Data are expressed as median [interquartile range] or frequency (%). BMI, body mass index; ES, effect size.

**Table 4:** Factors related to the severity of dysmenorrhea in athletes.

	B	Exp(B)	95% CI		*p*
Age (years)	0.123	1.130	0.977	1.308	0.10
BMI (kg/m^2^)	0.051	1.052	0.968	1.144	0.23
Sleeping hours (hours)	−0.047	0.954	0.832	1.094	0.50
Skipping meals (yes)	0.386	1.471	0.957	2.262	0.07
Age at menarche (years)	−0.160	0.852	0.765	0.950	<0.01
Menstrual cycle (days)	−0.018	0.982	0.956	1.009	0.19
Menstrual period (days)	0.269	1.309	1.154	1.484	<0.01
Training hours(hours/week)	0.026	1.026	1.004	1.048	0.02
Competition level (low)	0.117	1.124	0.923	1.368	0.25

BMI, body mass index; CI, confidence interval; Exp(B), odds ratio.

**Table 5:** Factors related to the severity of dysmenorrhea in non-athletes.

	B	Exp(B)	95% CI		*p*
Age (years)	0.115	1.122	0.908	1.386	0.29
BMI (kg/m^2^)	−0.066	0.936	0.834	1.052	0.27
Sleeping hours (hours)	−0.067	0.935	0.743	1.177	0.57
Skipping meals (yes)	0.170	1.185	0.665	2.114	0.57
Age at menarche (years)	−0.119	0.888	0.758	1.040	0.14
Menstrual cycle (days)	−0.044	0.957	0.918	0.997	0.04
Menstrual period (days)	0.355	1.426	1.161	1.752	<0.01
Exercise hours (hours/week)	−0.067	0.935	0.843	1.038	0.20

BMI, body mass index; CI, confidence interval; Exp(B), odds ratio.

## Data Availability

The data presented in this study are not publicly available in compliance with the investigation confidentiality and are available from the corresponding author on reasonable request.

## References

[1] Sultan C., Gaspari L., Paris F. (2012). Adolescent dysmenorrhea. Endocr. Dev..

[2] Zaiei S., Faghihzadeh S., Sohrabvand F., Lamyian M., Emamgholy T. (2001). A randomised placebo-controlled trial to determine the effect of vitamin E in treatment of primary dysmenorrhoea. BJOG.

[3] Nohara M., Momoeda M., Kubota T., Nakabayashi M. (2011). Menstrual cycle and menstrual pain problem and related risk factors among Japanese workers. Ind. Health.

[4] Ameade K.P.E., Amalba A., Mohammed S.B. (2018). Prevalence of dysmenorrhea among university students in Northern Ghana, its impact and management strategies. BMC Women’s Health.

[5] Soderman L., Edlund M., Marions L. (2019). Prevalence and impact of dysmenorrhea in Swedish adolescent. Acta Obstet. Gynecil. Scan..

[6] Lacovides S., Avidon I., Bentley A., Baker F. (2014). Reduced quality of life when experiencing menstrual pain in women with primary dysmenorrhea. Acta Obstet. Gynecil. Scan..

[7] Quick F., Mohammad-Alizadeh-Charandab S., Mirghafourvand M. (2019). Primary dysmenorrhea with and without premenstrual syndrome: Variation in quality of life over menstrual phases. Qual. Life Res..

[8] Kazama M., Maruyama K., Nakamura K. (2015). Prevalence of dysmenorrhea and Its correlating lifestyle factors in Japanese female junior high school students. Tohoku J. Exp. Med..

[9] Hashim T.R., Alkhalifah S.S., Alsalman A.A., Alfaris D.M., Alhussaini A.M., Qasim R.S., Shaik S.A. (2020). Prevalence of primary dysmenorrhea and its effect on the quality of life amongst female medical students at King Saud University, Riyadh, Saudi Arabia. Saudi Med. J..

[10] Qin L.-L., Hu Z., Kaminga A.C., Luo B.-A., Xu H.-L., Feng X.-L., Liu J.-H. (2020). Association between cigarette smoking and the risk of dysmenorrhea: A meta-analysis of observational studies. PLoS ONE.

[11] Ju H., Jones M., Mishra G. (2014). The prevalence and risk factors of dysmenorrhea. Epidemiol. Rev..

[12] Wang L., Wang X., Wang W., Chen C., Ronnennberg A.G., Guang W., Huang A., Fang Z., Zang T., Wang L. (2004). Stress and dysmenorrhoea: A population based prospective study. Occup. Environ. Med..

[13] Rafique N., Al-Sheikh H.M. (2018). Prevalence of menstrual problems and their association with psychological stress in young female students studying health sciences. Saudi Med. J..

[14] Khotimah K., Jauzak R.R.R.A., Nurunniyah S., Maharani O., Wahyuningsih W. (2020). Association of BMI and Sport activity habits with dysmenorhea. J. Ners Dan Kebidanan Indones..

[15] Armour M., Parry K.A., Steel K., Smith C.A. (2020). Australian female athlete perceptions of the challenges associated with training and competing when menstrual symptoms are present. J. Sports Sci. Coach..

[16] Kartal B., Kissal A., Kaya M. (2020). Comparison of Athletes and Sedentary Students in Terms of Premenstrual Syndrome and Dysmenorrhea. Ordu Univ. J. Nurs. Stud..

[17] Findlay J.R., Marcrae H.E., Whyte Y.I., Easton C., Forrest J.L. (2020). How the menstrual cycle and menstruation affect sporting performance: Experiences and perceptions of elite female rugby players. Br. J. Sports Med..

[18] Homai H.M., Shafai F.S., Zoodfekr L. (2014). Comparing menarche age, menstrual regularity, dysmenorrhea and analgesic consumption among athletic and non-athletic female students at universities of Tabriz-Iran. Int. J. Women’s Health Reprod. Sci..

[19] Bourdel N., Alves J., Pickering G., Ramilo I., Roman H., Canis M. (2015). Systematic review of endometriosis pain assessment: How to choose a scale?. Hum. Reprod..

[20] Arafa A.E., Senosy S.A., Helmy H.K., Mohamed A.A. (2018). Prevalence and patterns of dysmenorrhea and premenstrual syndrome among Egyptian girls (12–25 years). Middle East. Fertil. Soc. J..

[21] Tomczak M., Tomczac E. (2014). The need to report effect size estimates revisited. An overview of some recommended measures of effect size. TRENDS Sport Sci..

[22] Polat A., Celik H., Gurates B., Kaya D., Nalbant M., Ebru K., Hanay F. (2009). Prevalence of primary dysmenorrhea in young adult female university students. Arch. Gynecol. Obset..

[23] Ortiz I.M., Rangel-Flores E., Carrillo-Alarcón C.L., Veras-Godoy A.H. (2009). Prevalence and impact of primary dysmenorrhea among Mexican high school students. Int. J. Gynaecol. Obstet..

[24] Bavil A.D., Dolatian M., Mahmoodi Z., Baghban A.A. (2019). A comparison of physical activity and nutrition in young women with and without primary dysmenorrhea [version 1, peer review: 2 approved, 1 approved with reservations]. F1000Research.

[25] Tsai S.-Y. (2016). Effect of yoga exercise on premenstrual symptoms among female employees in Taiwan. Int. J. Environ. Res. Public Health.

[26] Motahari-Tabari N., Shirvani A.M., Alipour A. (2017). Comparison of the effect of stretching exercises and mefenamic acid on the reduction of pain and menstruation characteristics in primary dysmenorrhea: A randomized clinical trial. Oman Med. J..

[27] Zeinab K.A., Mohamadreza T.M., Alireza J.K. (2017). The effects of pilates exercise and careway supplementation on the levels of prostaglamdin E2 and perception dysmenorrhea in adolescent girls non-athlete. Asian Exerc. Sport Sci. J..

[28] Dehnavi M.Z., Jafarnejad F., Kamali Z. (2018). The Effect of aerobic exercise on primary dysmenorrhea: A clinical trial study. J. Educ. Health Promot..

[29] Czajkowska M., Drosdzol-Cop A., Galazka I., Naworska B., Skrzypulec-Plinta V. (2015). Menstrual cycle and the prevalence of premenstrual syndrome/premenstrual dysphoric disorder in adolescent athletes. Pediatr. Adolesc. Gynecol..

[30] Kitamura M., Takeda T., Koga S., Nagase S., Yaegashi N. (2012). Relationship between premenstrual symptoms and dysmenorrhea in Japanese high school students. Arch. Womens Ment. Health..

[31] Pascoe M.C., Thompson D.R., Ski C.F. (2017). Yoga, mindfulness-based stress reduction and stress-related physiological measures: A meta-analysis. Psychoneuroendocrinology.

[32] Casey M.L., MacDonald P.C., Mitchell M.D. (1985). Despite a massive increase in cortisol secretion in women during parturition, there is an equally massive increase in prostaglandin synthesis. A paradox?. J. Clin. Investig..

[33] Febbraio M.A. (2007). Exercise and inflammation. J. Appl. Physiol..

[34] Fernandez-Martinez E., Onieva-Zafra M.D., Parra-Fernandez M.L. (2018). Lifestyle and prevalence of dysmenorrhea among Spanish female university students. PLoS ONE.

[35] Kural M.R., Noor N.N., Pandit D., Joshi T., Patil A. (2015). Menstrual characteristics and prevalence of dysmenorrhea in college going girls. J. Family Med. Prim. Care.

